# Fabrication of Meso-Porous Sintered Metal Thin Films by Selective Etching of Silica Based Sacrificial Template

**DOI:** 10.3390/nano4030686

**Published:** 2014-08-04

**Authors:** Ludovic F. Dumée, Fenghua She, Mikel Duke, Stephen Gray, Peter Hodgson, Lingxue Kong

**Affiliations:** 1 Institute for Frontier Materials, Deakin University, Pigdons Road, Waurn Ponds 3216, Victoria, Australia; E-Mails: mary.she@deakin.edu.au (F.S.); peter.hodgson@deakin.edu.au (P.H.); lingxue.kong@deakin.edu.au (L.K.); 2 Institute for Sustainability and Innovation, College of Engineering and Science, Victoria University, Hoppers Lane, Werribee 3030, Victoria, Australia; E-Mails: mikel.duke@vu.edu.au (M.D.); stephen.gray@vu.edu.au (S.G.)

**Keywords:** meso-porous metal materials, metal nano-particle sintering, surface texturing, metal particle coalescence, silica template etching

## Abstract

Meso-porous metal materials have enhanced surface energies offering unique surface properties with potential applications in chemical catalysis, molecular sensing and selective separation. In this paper, commercial 20 nm diameter metal nano-particles, including silver and copper were blended with 7 nm silica nano-particles by shear mixing. The resulted powders were cold-sintered to form dense, hybrid thin films. The sacrificial silica template was then removed by selective etching in 12 wt% hydrofluoric acid solutions for 15 min to reveal a purely metallic meso-porous thin film material. The impact of the initial silica nano-particle diameter (7–20 nm) as well as the sintering pressure (5–20 ton·m^−2^) and etching conditions on the morphology and properties of the final nano-porous thin films were investigated by porometry, pyknometery, gas and liquid permeation and electron microscopy. Furthermore, the morphology of the pores and particle aggregation during shear mixing were assessed through cross-sectioning by focus ion beam milling. It is demonstrated that meso-pores ranging between 50 and 320 nm in average diameter and porosities up to 47% can be successfully formed for the range of materials tested.

## 1. Introduction

Nano-porous metal frameworks exhibit unique surface to volume ratios whereby the metal natural’s optical, catalytic and mechanical properties become dramatically enhanced [1,2]. Typically, through sufficient increase of the relative density of metal nano-particles (NPs) assembled across an otherwise porous matrix, non-linear optical responses a decade higher than that predicted by effective medium theories were obtained [3]. Similarly, partially coalesced metal NPs offer thermal and electrical conductivities significantly higher than bulk metal [4]. Metal nano-textured surfaces and meso-porous networks are emerging as advanced platforms for chemical reactions, sensing and separation [5].

Current commercial porous metal materials have limited scope due to their relatively large micron sized pore inherent to their fabrication process [5]. To date, commercial porous metal materials are either processed by sintering of macro sized metal particles or foaming. Sintering consists of the hot-compression of metal powders or fibres at the softening temperature of the metal to form a semi-porous network. Alternatively, foaming is performed by combustion of foaming agents within molten metals leading to the release of gases to form macro-cavities and pores within the cooling cast metal slab [6]. These fabrication processes typically lead to large pore size distributions (>1 µm), low pore connectivity and limited porosity (<45%), and do not offer scope for the processing of nano-structured surfaces with enhanced surface properties reported for finer nanoscale metal frameworks [7]. Recently, metal nano-foaming, electro-spinning and metal electro-less deposition were applied to the development of novel meso-porous electrodes, heat sinks or separation materials [8,9,10,11]. These techniques offer the advantage of very fine and highly controllable pore formation across metal materials and up-scalable, large surface areas [5,10]. The surface roughness, specific surface area and topography of these materials can also be tuned by varying the processing parameters leading to different ranges of metal grain size distributions and thus to adjustable surface energies with potential benefits in catalysis due to the high reactivity of most nano-structured metal materials [12].

However, the most notable recent progress has been achieved through a deeper understanding of coalescence mechanisms and the development of advanced routes to stabilize metal NPs into ordered and porous crystalline micro-network [13]. Further improvements of the stability of long range ordered metal NP assemblies and enhancement of pore density and homogeneity are still, however, pressing issues and would lead to more controlled metal nano-fabrication routes [5]. Powder metallurgy and nano-metallurgy, which relates to the controlled sintering of metal powder and nano-powder mixtures, are emerging research fields for the processing of nano and meso porous metal materials [14,15]. However, issues related to powder mixing, including spontaneous phase separation of the powders and de-mixing during powder flow are still only partially understood phenomenon. The size, but also the density and shape of the multiple distributions of mixed particles were shown to lead to near complete lamellar type phase separation thus offering new routes to the processing of highly porous layered metal materials [16,17,18]. In addition, the controlled coalescence of metal NPs is also a challenge due to the naturally high surface energy of the materials leading to rapid densification. It is, therefore, critical to investigate ways to limit coalescence mechanisms in order to maintain porosity of the nano-structure upon removal of the sacrificial phase.

In this paper, a new and simple route to process stable meso-porous metal materials by cold-pressing of mixed powders and selective etching of a sacrificial matrix will be demonstrated. This technique is applied to the processing of pure silver and copper meso-porous thin films. The potential of the technique to lead to stratified materials with a range of porosities and pore size distributions will be critically analyzed in light of the powder mixture compositions and morphologies and properties of final meso-porous materials. In addition, potential applications of meso-porous metal materials will be discussed.

## 2. Results and Discussion

**Figure 1:**
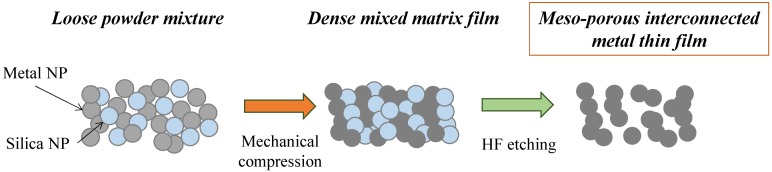
Schematic of the powder mixing, cold-compression and silica etching process leading to a meso-porous pure metal material.

The formation of the meso-porous metal thin films by cold-pressing of mixed NP powder mixtures is depicted in Figure 1. The NPs were mixed together and poured into the die and the powder levelled through tangential vibrations prior to cold-pressing. The mixture of the two powders, while Si and dark grey Ag, were gently but thoroughly mixed until formation of a homogeneous light grey coloured powder.

**Figure 2:**
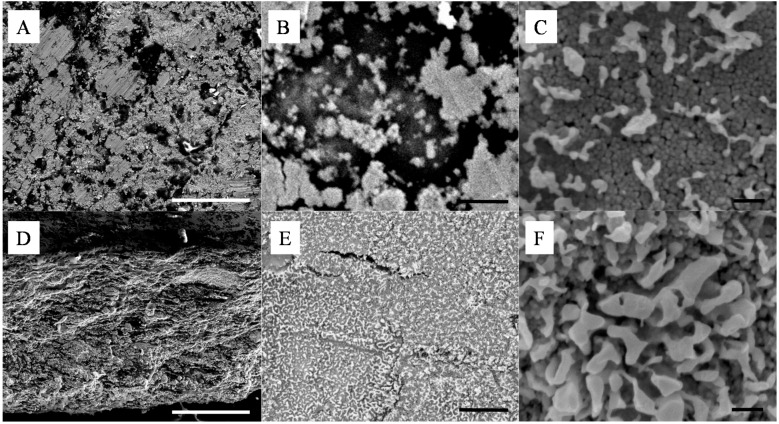
Representative SEMs of the silica - silver cold-pressed composites at 20 ton·m^−2^; (**A**–**C**) are surface and (**D**–**F**) cross section images of a 10 wt% silica content composite. The scale bars of (**A**,**D**), (**B**,**E**) and (**C**,**F**) respectively represent 100 μm, 1 μm and 100 nm.

Although powder mixing was thorough and no aggregates could be detected by optical microscopy and visual inspections of the powders, this process, as shown in Figure 2A–D, lead to the formation of dense materials with apparent particle segregation domains on the surface and across the thickness. The formation of layered NPs sub-structures is particularly visible in Figure 2D. De-mixing of powders is a process which was previously related to the size, density and shape of the mixed particles. The moving particles flowing across a vortex or a Couette flow may phase separate through simple gravity separation [14] and the smaller particles may then form a bed and infiltrate gaps between the larger particles. This phenomenon can be enhanced upon mechanical agitation of the mixed powders creating movement of the larger particles and facilitating the displacement of the smaller particles. Segregation induced separation has not been reported to date for sub-100 nm particles and has implications for the dry processing of NP powders (Figure S1). Here, the clustering of the particles and their aggregation may also be enhanced from surface affinities between the high surface energy NPs [1]. Although at the nanoscale Si and Ag materials shall be oppositely charged, SiO_2_ and Ag being respectively naturally negatively and positively charged due to surface valence deficiency, the de-mixing may be caused by surface frictions between the like particles, leading to the formation of the visible clusters from the material surface [19]. These frictions may lead to electrostatic interactions between particles upon flowing and may therefore explain some of the clusters formation [20]. The impact of compression pressure on the sintering was first investigated. Interestingly, compression pressures lower than 20 ton·m^−^^2^ did not lead to well-structured and mechanically stable thin films and a minimum energy was required to induce sintering of the Ag particles to form strong networks of metal at silica contents larger than 5 wt%, corresponding to ~42 v% (Figure S3). Therefore all samples presented in this work were cold-pressed at 20 ton·m^−2^.

**Figure 3:**
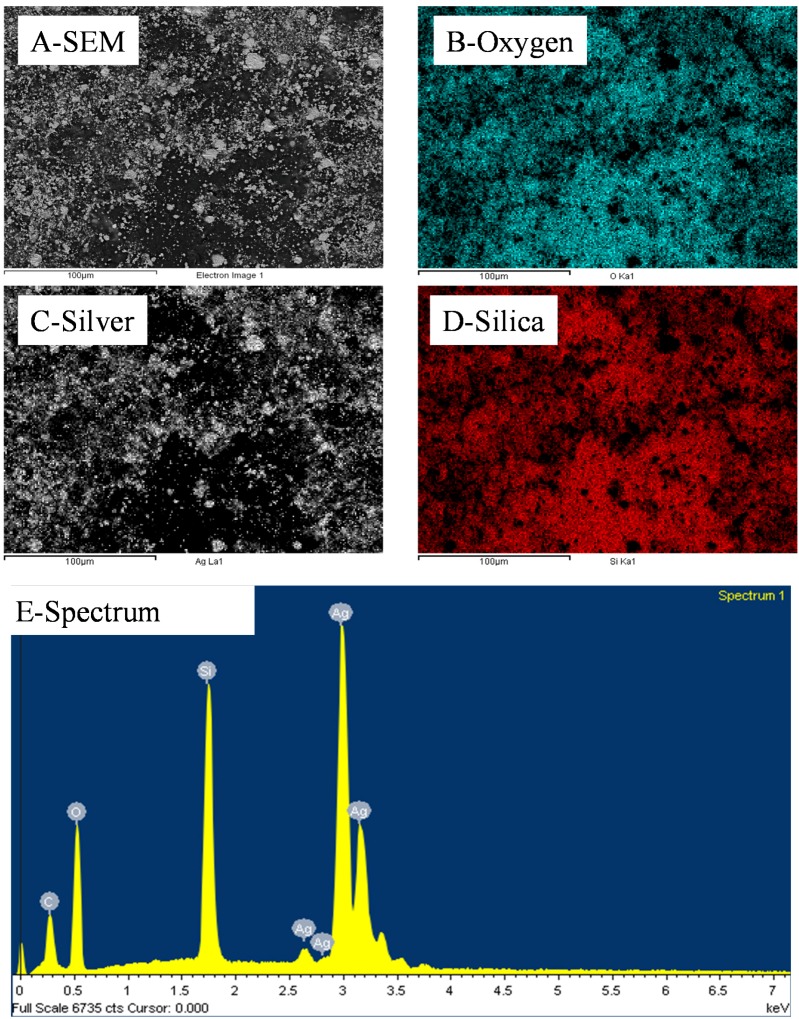
Representative EDS distributions of Si, Ag and O elements across a 10 wt% silica content cold-pressed composite before silica etching presented in Figure 1—carbon is present due to the sputtering step for sample preparation for SEM imaging.

The size of the surface clusters was found by SEM analysis to range, across all studied samples, between 500 nm and 2 μm (Figure 2A). Interestingly, the clusters seem to be larger on the surface of the material than across its thickness as seen in Figure 2E,F. There, after cold-compression, the average size of the clusters was found to be reduced to approximately 100 nm, corresponding to the agglomeration of ~5 Ag NPs. It is clear from these cross section micrographs that the size of the corresponding silica clusters is also close to the same dimensions, with up to 10^12^ silica NPs per cluster. This particle distribution was confirmed by energy dispersive spectroscopy (EDS) mapping (Figure 3) where the clusters could be clearly attributed to either nearly pure Ag or SiO_2_. Furthermore small angle x ray scattering (SAXS) analysis of the samples before etching shows a large particulate distribution between 5 and 35 nm characteristic of the SiO_2_ particles and small clusters (Figure S2).

As seen in Figure 2, the energy brought to the system by cold-pressing lead to the near complete coalescence of the soft Ag NPs and the formation of clear and well interconnected bridges across the silica matrix. The ability for a metal to coalesce at low input energy, such as the system described here, is largely related to the surface energy of the metal and to localized vacancies on the surface of the particles allowing for metal grain deformation and ultimately re-conformation and fusion [21] similar to that previously reported for solution particle formation and coalescence [22]. On the other hand, the hard SiO_2_ spheres appeared undamaged even at compressing pressures of 20 ton·m^−2^ and particle clusters were only found on the surface and across the thickness of the composite thin films. It is possible that the application of the pressure led to particle movement across the powder bed which may favor segregation. The initial powder bed thickness was estimated, based on the volume of particles poured in the dye, to be around 250 μm, while the final thickness of the compressed thin film samples was measured to be around 25 μm. This compression rate of the bed is therefore close to 90% suggesting large displacements of the nanoscale particles during the early stage of the cold-pressing process. The total energy of the particles can be divided into three main components, namely their kinetic energy, their potential energy and their surface energy. Although the kinetic energy of the particles is here close to nil due to the compression leading to a nearly static, low velocity, system [23], the smaller particles potential energy may be reduced by moving across the mixed bed particles prior to Ag coalescence [22]. This may favor segregation by gravimetric sorting of the particles. In addition, the strong friction between the particles upon compression will lead to localized heating and favor sintering and surface grain aggregation [21]. The soft Ag particles will tend to limit their surface energy by coalescence and will expand their overall volume by combining with neighboring like particles [24]. This process, closely related to powder metallurgy, may be used for the preparation of metal alloys from different metal NP mixtures by cold-pressing.

**Figure 4:**
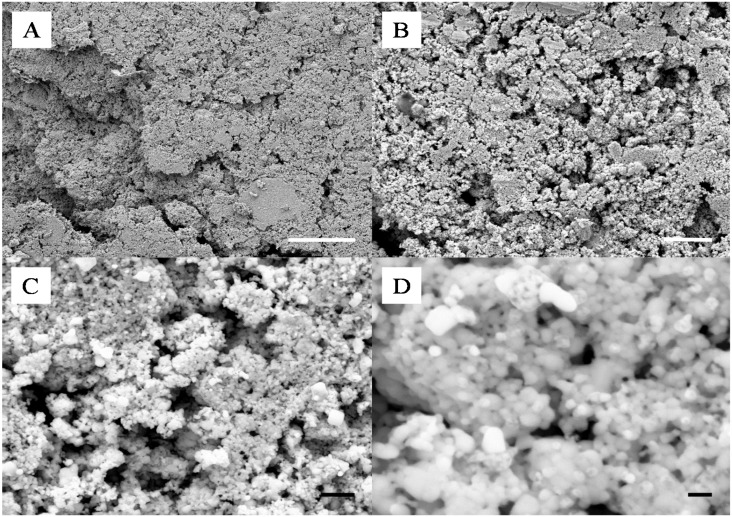
Representative SEMs of the surface of an etched 10 wt% SiO_2_-Ag composite similar to those presented in Figure 1; scale bars of (**A**), (**B**), (**C**) and (**D**), respectively correspond to 100 μm, 10 μm, 1 μm and 100 nm.

**Figure 5:**
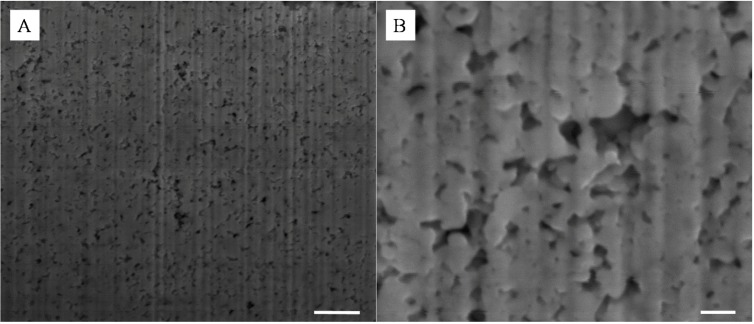
Representative SEMs of the cross-section of an etched 10 wt% SiO_2_-Ag composite; scale bars of (**A**) and (**B**) are 1 μm and 200 nm respectively. The images were taken approximately 10 μm from the surface.

**Figure 6:**
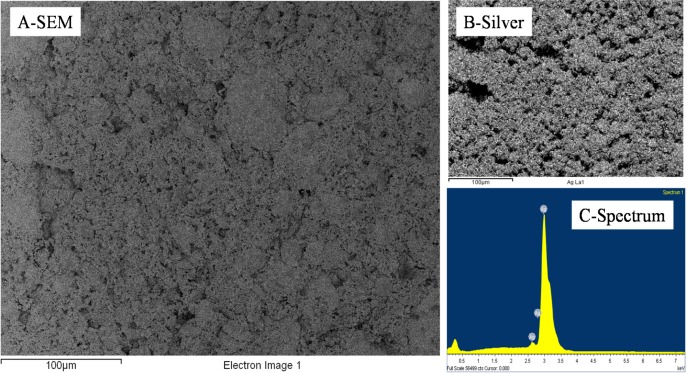
Representative EDS distributions of Si, Ag and O elements across a 10 wt% silica content cold-pressed composite after silica etching–a small amount of carbon can be detected residual from sample preparation for SEM imaging; no Si and minutes amount of O could be detected; the scale bar corresponds to 100 μm.

As seen in Figure 4, the Ag sintered clusters were very stable upon etching in hydrofluoric acid (HF) and lead to the formation of pore domains in the site of the SiO_2_ clusters and NPs. The presence of SiO_2_, potentially remaining from the etching process was also checked by EDS, but, as seen in Figure 5 and Figure 6, none could be detected after the etching process and the structure was found to be made of pure Ag.

**Figure 7:**
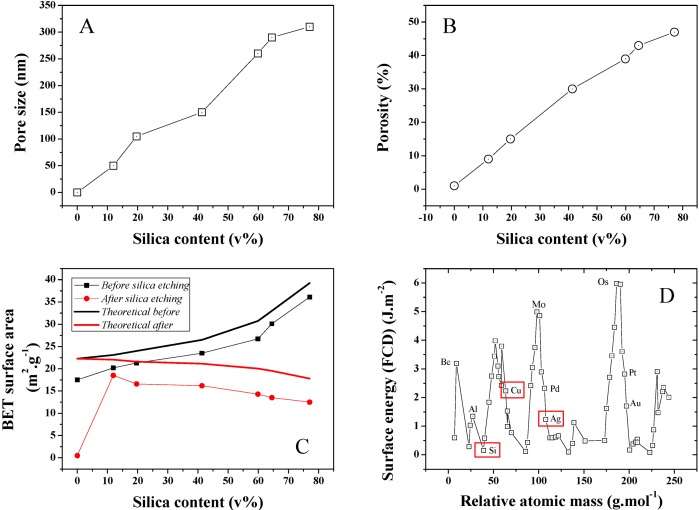
(**A**) Average pore size distribution obtained for the series of Ag-Si nano-composites after Si etching; (**B**) Average porosity for the same series; (**C**) BET surface area for the series of samples before and after etching and theoretical BET surface area calculated for an estimated individual surface area of the Si and Ag NP powders of 23.81 and 30 m^2^·g^−1^. The surface area of the cold press sample at 0 wt% of SiO_2_ was extremely low and thus 0 due to strong particle coalescence; and (**D**) FTD calculated surface energy for the main elements of the periodic table and visual representation of the position of Si, Cu and Ag. The 0 wt% silica data points for pore size and porosity were extrapolated since no data could be obtained neither by perm-porometry nor pyknometry.

The average pore size distribution of a series of different silica loading samples was investigated and is presented in Figure 7A. Pore size is found to steadily increase with silica content during the isobaric compression, suggesting an increase of the clustering due to stronger interactions between the SiO_2_ NPs during cold-pressing. This was confirmed by SEMs taken on Focus Ion Beam (FIB) cross-sectioned samples (Figure 5) where 50–400 nm diameter pores could be detected. The average pore size for 10 and ~80 v% samples was found to increase nearly 6 fold from 50 to 300 nm (Conversion wt% to v% is presented in Figure S3). Furthermore, porosity was also found to strongly increase upon SiO_2_ content increase in the initial powder bed. A typical pore size distribution for a 12 v% SiO_2_ content sample is shown in Figure S4. As seen in Figure 7B, porosity increased for the 10 and ~80 v% samples from approximately 10% to 47%. This strong discrepancy between the SiO_2_ volume and the final porosity may be explained by the larger SiO_2_ clusters visible on the sample surface before etching and from potential recombination of the Ag matrix upon HF etching. Both the surface area and thickness of the samples before and after etching was found to shrink by about 10%. It is, however, challenging to properly measure this volume change due to the rough surface of the material, limiting the accuracy of any thickness measurement attempts and to potential decay on the side of the samples due to the presence of SiO_2_ clusters. This hypothesis was confirmed by BET surface area analysis of the series of samples, which was also found to steadily increase with SiO_2_ content (Figure 7C). The maximum specific surface area of the samples were calculated based on that of the individual particles before compression. A discrepancy between these values and that measured is found, which is here related to the strong surface energy and roughness change of the silver due to the plastic deformation leading to grain deformation and ultimately at an excitation energy high enough coalescence. The reactivity of metal surface is illustrated in Figure 7D where modelled surface energies for a range of elements were computed [25].

**Figure 8:**
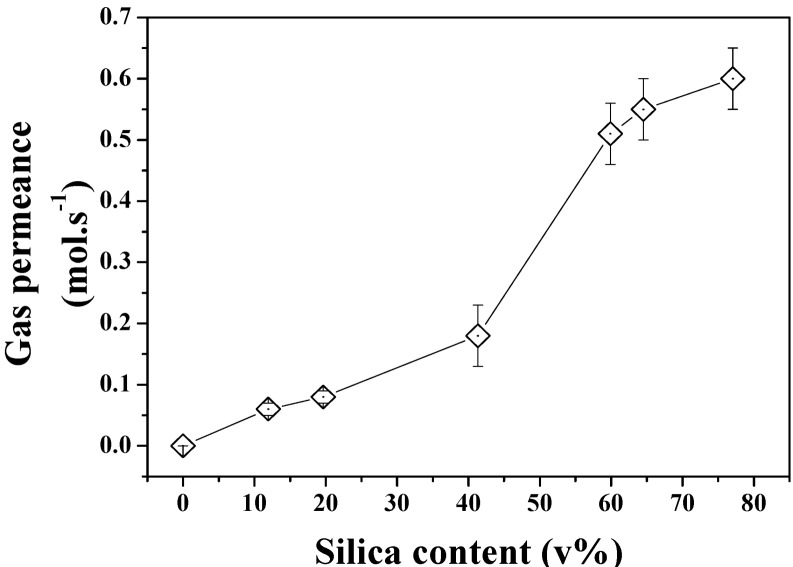
N_2_ permeance across the etched Si-Ag NP composites.

The gas permeation properties of the same series of samples were also investigated to demonstrate the formation of true through pores across the structures. As seen in Figure 8, the increasing pore size and porosity lead to enhanced gas permeation. Interestingly, a stronger shift was found to occur between 40 and 60 SiO_2_ v% content in the powder beds which could be related to the formation of larger defects on the edge of the samples or to macro voids across the materials which cannot be otherwise characterized by direct pore size or porosity measurements. The break-up of the gas permeation at this level could be related to localized inhomogeneity of the materials and variations of relative pore size and porosity despite the care taken in both sample preparation and tests. The relationship between pore size and gas permeation is expected to be linear for this regime of diffusion at constant porosity. However the increasing porosity directly related to pore size and demonstrated in Figure 7 may affect this trend and lead to non-linear gas permeation regimes due to great variations of structures between the samples. Further analysis of the formation of the clusters and pores upon etching for Ag are required and shall be carried out in order to process narrower pore size distribution materials and process materials with fixed porosity and ranging pore size distributions.

**Figure 9:**
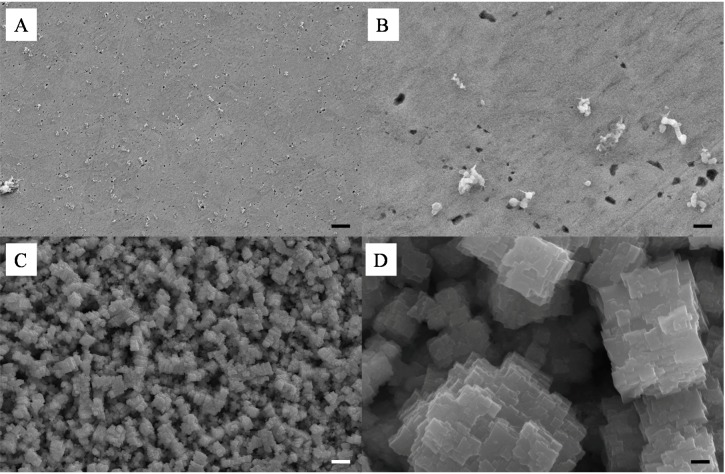
Representative SEMs of the surface of a 10 wt% Si-Cu composite before (**A**,**B**) and after (**C**,**D**) HF etching; scale bars of (**A**,**C**) and (**B**,**D**) respectively correspond to 2 μm and 200 nm.

A similar study was performed with SiO_2_–Cu NPs. This was performed to assess the feasibility of the technique to produce lower cost meso-porous metal materials. Most of the work reported to date on the coalescence of metal NPs has focused on the use of noble and inert metals such as Ag, Gold and Platinum. The Cu particles must be handled in an inert atmosphere in order to avoid their spontaneous combustion in contact with O_2_ gas. For this reason only a select number of samples were prepared and etched in HF following the same protocol as for the Ag NPs. As seen in Figure 9A–D, the formation of the composite was in essence similar to that of the SiO_2_-Ag frameworks, although smaller aggregates were found on the surface of the samples upon compression. The surface of the material was also found to be much smoother than that of the SiO_2_-Ag composites, which was attributed to the higher surface energy of Cu NPs and thus their stronger capability to coalesce. Etching of the composite samples led to the formation of very different pore shapes than those obtained for Ag films. A change of solution color during etching, from transparent to green, suggested a competitive etching between the silica and the copper. This was confirmed by SEM as seen in Figure 9C,D, where cubic aggregates can be seen. Interestingly it appears that the surface of the coalesced Cu NPs was partially corroded which lead to a much rougher and more ordered surface with clear cubic particles formation. Clear pores can be seen from the SEMs suggesting a similar network formed across the pure copper clusters. Although highly promising more analysis should be carried out to fully reveal the properties of these Cu based meso-porous structures.

## 3. Experimental Section

### 3.1. Materials and Powder Preparation

Commercial silica NPs of 7 nm and metal NPs of 20–25 nm in diameter were purchased from Sigma Aldrich and NanoAmor (US) respectively. Both silver and copper NPs with a 0.2 wt% poly(vinyl pyrollidone (PVP) coating were purchased for this study and used as received without further treatments. The particles were weighed on a 10^−5^ g accuracy balance and first gently hand-mixed together in a mortar. The silica ratio was varied between 0 and 100 wt% to assess the impact of the sacrificial phase on the material morphology. Then the mixture was placed in plastic test tubes and vigorously shaken for 5 min on a Vortex 1 shaker. Samples of the powders were then examined under an optical microscope (50× on an Olympus DP70 Digital Microscope Camera, Münster, Germany) and appeared well mixed and free of aggregates.

The powders were mechanically compressed to induce localized sintering between adjacent metal based NPs. Fixed mass of powders were gently poured into a 13 mm diameter die and cold-pressed at room temperature and pressures comprised between and 5–20 ton·m^−2^ for 5 min. After pouring the powder mixture in the die to form a particle bed, the die was gently vibrated for approximately 5 s on a Vortex 1 shaker to homogenize the bed thickness. The silica NPs were then removed by HF etching. A 12 wt% HF/water solution was used and samples were exposed for 15 min to the etching solution.

### 3.2. Characterization Methods

Scanning Electron Micrographs (SEMs) and EDS results were obtained on a dual beam Gallium (Ga) FIB Quanta FEI SEM fitted with an EDAX sensor for EDS analysis. The working distance was 10 mm and the accelerating voltage for imaging and elemental mapping were 5 and 20 keV respectively. Milling with the Ga FIB was performed at 20 keV and in 3 steps, including a rough milling step at 14 nA and two cleaning steps at 1 and 0.3 nA. Samples were coated with carbon prior to image analysis. Image analysis and statistics were performed with GIMP 2.8.

An average BET surface area was determined by N_2_ adsorption on a Micromeritics Tristar 3000. The samples were first degassed for 70 h at 120 °C and then analyzed at 77 K. Pore size distributions were determined by perm-porometry, on a gas flow porometer from Porous Materials Inc. (Ithaca, NY, USA), in wet-up/dry-up configuration following a procedure previously described [26]. A Galwick solution was applied to wet the top surface and the sample pressurized under analytical grade N_2_. An AccuPyc II 1340 Gas Displacement Density Analyzer from Micromeritics was used to evaluate the density of samples before and after etching. A Meso cell was used for the relative density measurements and He was used as an adsorption gas over a range of pressure from 0.1 to 1 atm. Disks of 5 mm in diameter were processed by cold pressing and their thickness measured with a micrometer to obtain the global volume of the sample. The thickness was fixed at 25 μm (± 3 μm) by controlling the amount of powder used for the cold compression tests. The procedure was previously detailed in [27].

SAXS experiments were performed on the SAXS/WAXS beamline at the Australian Synchrotron. The high brilliance and coherence of the beam allowed for very short acquisition time (300 ms). The energy of the beam was set at 11 keV and scattering from the Kapton tape was determined independently and removed as background from the signals. The camera length of this series of test was 0.6 m and the wavelength of the collimated beam was 1.0332 Å beam energy of 9.8 keV. The size of the x ray beam was 150 × 150 μm. The data were fitted with SAXSId after correction for the beam center position, pixel size and frame size. The integration was performed on the whole image except around the beam stop which was excluded. The small scattering angle, q, is inversely proportional to the scatterer diameter at small scattering angles as previously described in [28].

Gas permeance measurements were performed by placing the membrane in a 5 cm^2^ o-ring sealed holder which separates a 20 L upstream feed cylinder from a 50 mL permeate cylinder [29]. The feed vessel was isolated from both the vacuum and membrane holder, and filled with analytical grade N_2_ to 101 kPa with filtered and dry air. For testing, the permeate side was evacuated to a vacuum of 0.5 kPa, isolated from vacuum and the pressure allowed to rise once the isolating valve between the feed and permeate vessels was opened. The pressure of the permeate size was monitored over time until equilibrium was reached. The feed pressure remained essentially constant due to its much larger volume compared to that of the permeate. The permeance, f (mol·s^−1^), could then be determined by transposing the fitting of the pressure rise, P(t), in the permeate vessel following a procedure detailed in [29]. All the tests were performed at controlled temperature (20 ± 1 °C). The surface area of the membrane was comprised between 4 and 6 mm^2^ based on the size of the die used for cold-compression. The membranes were mounted onto a perforated gas impermeable poly(ethylene terephthalate) film and glued with an expoxy resin (Araldite 5 min quick cure).

## 4. Conclusions

Meso-porous pure silver and copper metal thin film materials were processed for the first time by selective etching of a silica based sacrificial template. The novel meso-porous materials were shown to exhibit homogeneous pore size distribution, porosity and specific surface area closely related to the content in silica of the composite film before etching. Discrepancies in pore size distributions were attributed to phase separation during powder pouring within the die and potential particle aggregation upon cold-pressing. Furthermore, it was demonstrated that full metal NP sintering can be achieved at room temperature only upon pressure application, opening the route to the controlled coalescence of NPs. Other template materials such as lyotropic crystals or polymeric beads could also be used as sacrificial materials to form complex 3D metal frameworks. Also the surface functionalization of the metal NPs could facilitate mixing with the silica template and lead to more ordered and stable meso-structures. Meso-porous metal materials, due to their high surface energy and high specific surface area, offer great promise for application in filtration, catalysis and sensing.

## Supplementary Materials

Supplementary File 1
