# Supplementary material for: Fabrication of Meso-Porous Sintered Metal Thin Films by Selective Etching of Silica Based Sacrificial Template
Source: Nanomaterials (Basel). 2014 Aug 4;4(3):686–99. doi: 10.3390/nano4030686 (PMC5304697; doi:10.3390/nano4030686)

Supplementary Materials

Figure S1. (**A**) SEM and (**B**) Representative EDS spectra and mapping of (**C**) Si, (**D**) O and (**E**) Ag elements across a fractured 10 wt% Si content cold-pressed composite before silica etching; the scale bar corresponds to 90 μm.

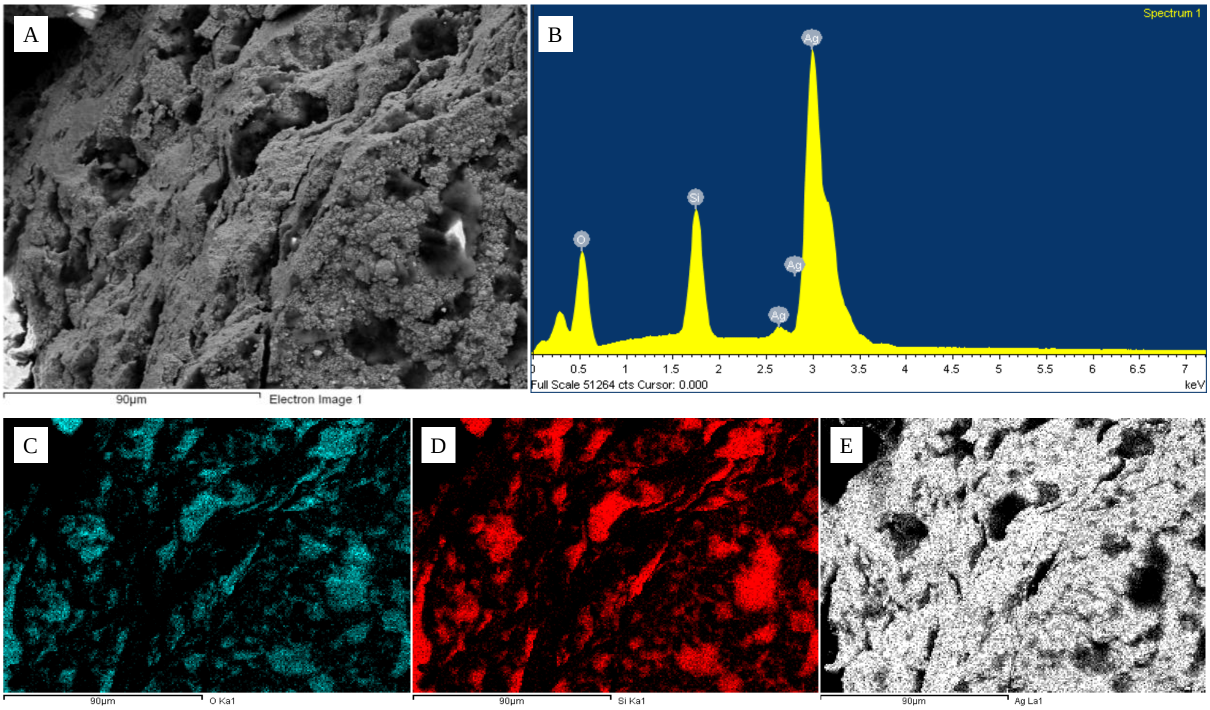

Figure S2. (**A**) integrated SAXS patterns for 10 and 20 wt% Si-Ag composites before and after etching and; (**B**) fit for the 10 wt% sample before etching log normal distribution with a dilute system structure factor and spheroid form factors; (**C**) residual trace for the modelling and; (**D**) size distribution for a log normal distribution showing a narrow distribution corresponding to the Si NPs. The samples after etching were shown to follow a Porod law suggesting.

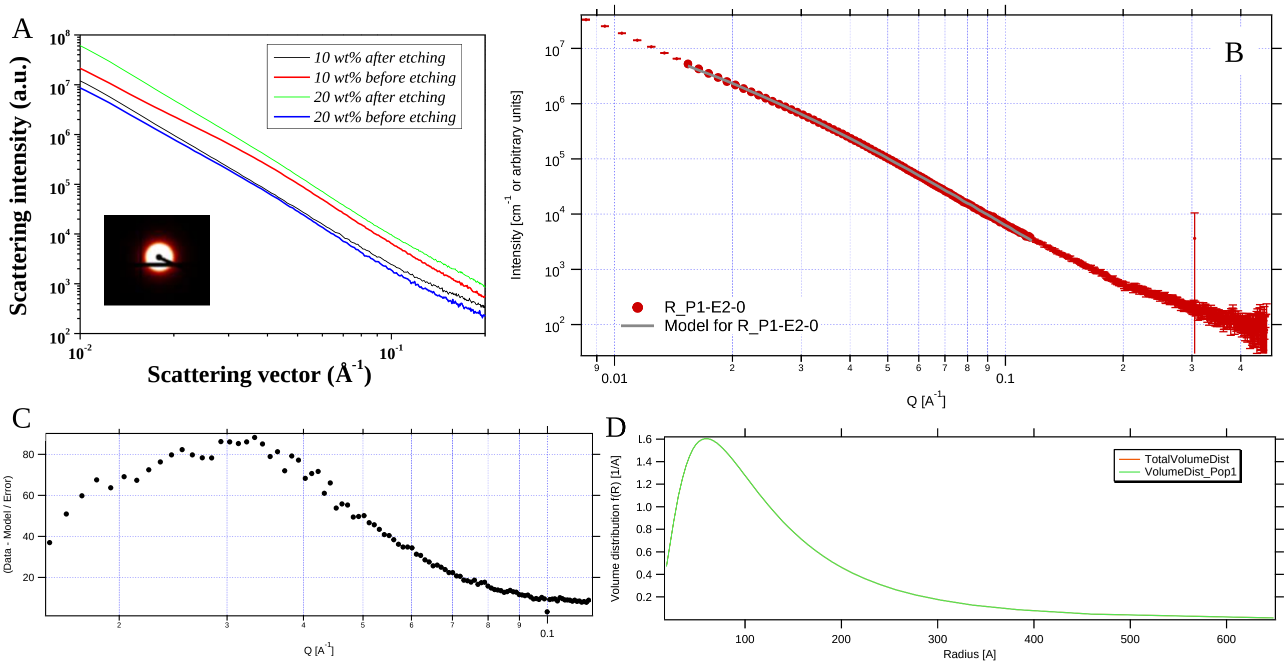

Figure S3. Relationship between weight % (wt%) and volume % (v%) for the silica silver mixtures.

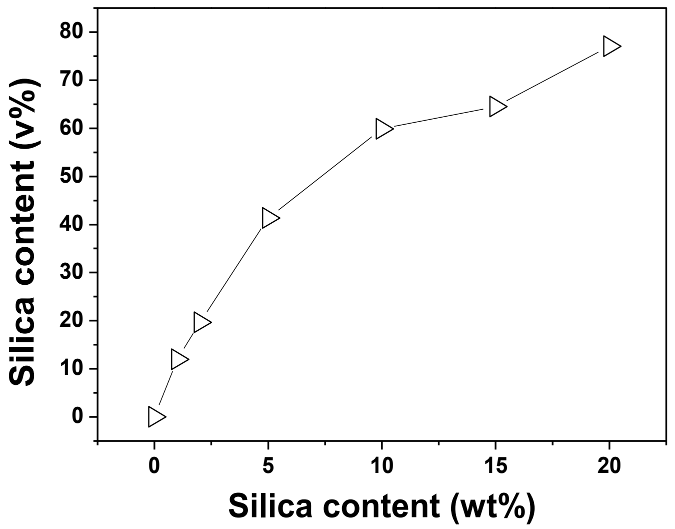

Figure S4. Pore size distribution for (**A**) 12, (**B**) 20, (**C**) 43 and (**D**) 62 v% different etched Ag thin films.

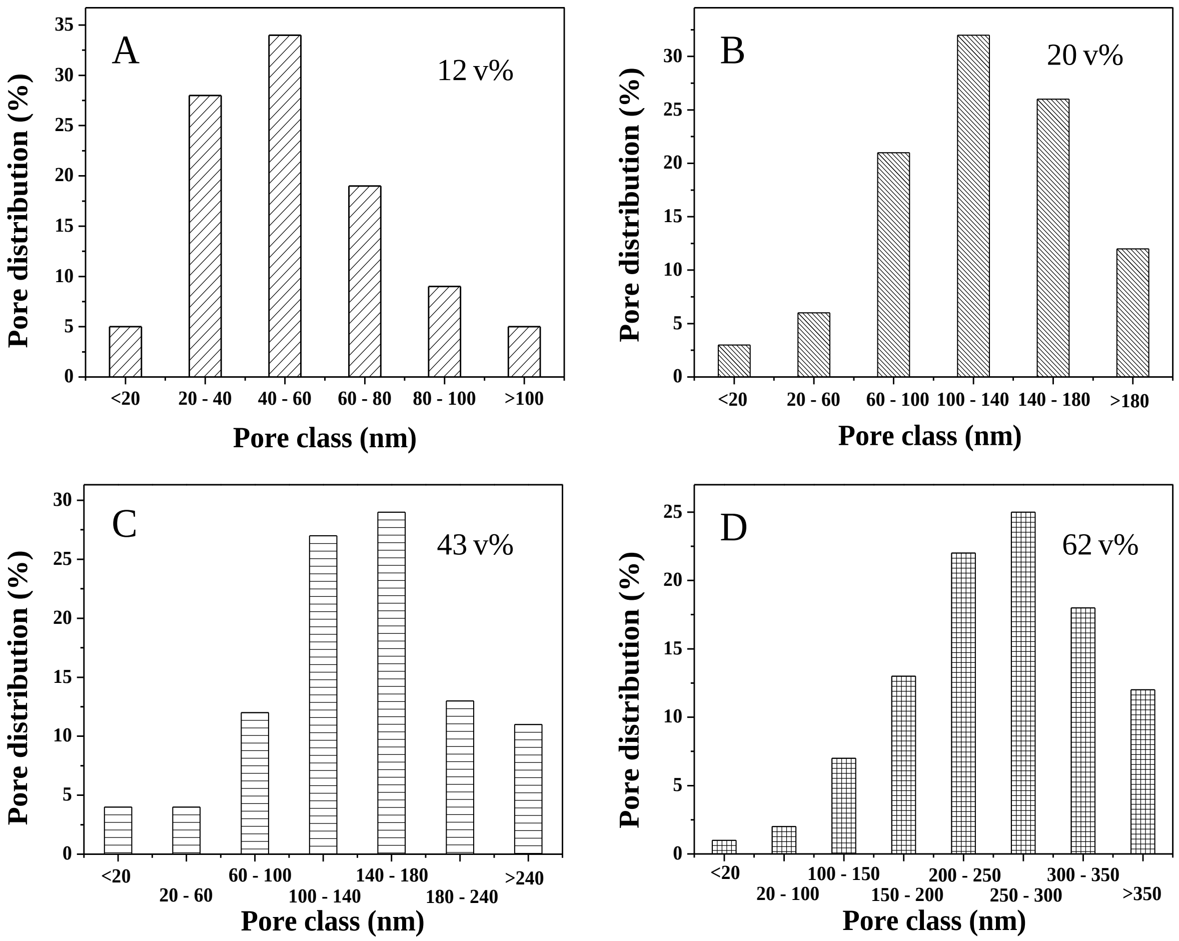

Supplement: Supplementary File 1 [file nanomaterials-04-00686-s001.pdf]
